# Stakeholders Perceptions of Barriers to Precision Medicine Adoption in the United States

**DOI:** 10.3390/jpm12071025

**Published:** 2022-06-22

**Authors:** Monica M. Schroll, Arushi Agarwal, Olivia Foroughi, Emily Kong, Omar Perez, Daryl Pritchard, Tom Breur, Kristen Garner Amanti, Gary Gustavsen

**Affiliations:** 1 Health Advances LLC, San Francisco, CA 94105, USA; aagarwal@healthadvances.com (A.A.); oforoughi@healthadvances.com (O.F.); emilymkong@gmail.com (E.K.); tbreur@healthadvances.com (T.B.); kgamanti@healthadvances.com (K.G.A.); ggustavsen@healthadvances.com (G.G.); 2 AstraZeneca, Wilmington, DE 19803, USA; omar.perez@astrazeneca.com; 3 Personalized Medicine Coalition, Washington, DC 20036, USA; dpritchard@personalizedmedicinecoalition.org

**Keywords:** precision medicine, precision oncology, utilization barriers, implementation challenges, healthcare professional (HCP) education, payer education, patient education

## Abstract

Despite evidence that precision medicine (PM) results in improved patient care, the broad adoption and implementation has been challenging across the United States (US). To better understand the perceived barriers associated with PM adoption, a quantitative survey was conducted across five stakeholders including medical oncologists, surgeons, lab directors, payers, and patients. The results of the survey reveal that stakeholders are often not aligned on the perceived challenges with PM awareness, education and reimbursement, with there being stark contrast in viewpoints particularly between clinicians, payers, and patients. The output of this study aims to help raise the awareness that misalignment on the challenges to PM adoption is contributing to broader lack of implementation that ultimately impacts patients. With better understanding of stakeholder viewpoints, we can help alleviate the challenges by focusing on multi-disciplinary education and awareness to ultimately improve patient outcomes.

## 1. Introduction

Precision medicine (PM), the practice of matching patients with the correct targeted therapy based on the patient’s biologic and molecular characteristics [1], has increasingly become the standard of care in cancer therapy since first reported more than 20 years ago [2]. The use of PM in oncology helps to ensure that patients receive effective treatments while minimizing potentially harmful side effects, which in turn increases quality of life and overall survival [3]. As the scientific community continues to understand the genetic underpinnings of cancer biology, the use of PM will continue to grow [4]. Outside of oncology, PM has started to grow more prevalent in areas such as autoimmune diseases and neurology [5,6] with the intent of improving the standard of care by removing the “trial and error” approach that has historically been employed.

The implementation of PM, especially biomarker testing, faces significant challenges. Some examples include practical limitations to PM utilization due to the adoption and implementation of novel biomarker testing and clinical decision support technologies and services, limited understanding of the clinical utility of biomarker tests to help guide patient management, and lack of consensus on levels of evidence necessary for the validation of particular biomarker tests as described by Lassen, et al. [7]. However, these challenges are positioned as stakeholder specific and often limited to the clinicians and/or patients [7]. Additional challenges to implementation include incorporating biomarker information into patient health records, delivering accessible and affordable PM technologies and services to patients, and ultimately contributing value for health care practitioners and payers [8]. The implementation of PM is difficult in the fragmented US health care system and many of the key stakeholders are often resistant to paradigm shifts in the standard of care [8]. Despite evidence that implementation of PM into daily health care delivery corresponds to benefits in patient care [9], there is work to be done in both the practices and policies that address the challenges at various levels of the healthcare system to fully integrate PM into the patient journey [7]. One of the overarching issues contributing to the relatively slow integration of PM for cancer care across all US healthcare systems is a lack of alignment on recognition of the challenges to PM implementation across key stakeholders, including payers and lab directors as well as clinicians and patients. A common understanding of the perceived barriers to PM will be valuable to inform community efforts to facilitate increasing the adoption of PM. 

To understand the perceived barriers to PM utilization in the US, a survey was conducted to gather the viewpoints of key stakeholders in the patient journey including surgeons, oncologists, lab directors, payers, and patients. To our knowledge, this is the first of its kind study that compares the viewpoints across these stakeholder groups and thus helps define the landscape of current implementation challenges, as well as a chance for the community to level set expectations.

The survey output revealed that there is a lack of recognition of the clinical utility of broad PM testing related to a lack of awareness and education amongst various health care stakeholders. Frequently, this led to medical policy coverages that were not aligned with clinician expectations nor with current National Comprehensive Cancer Network (NCCN) treatment guidelines or professional society testing guidelines. Additionally, the results of the survey highlighted discrepancies between stakeholder perceptions of patient education and awareness resources needed to facilitate PM including, utilization of patient support programs, genetic counseling, and availability of resources across the multi-disciplinary cancer care team. 

The lack of a common understanding of the clinical utility of PM and of available educational resources has contributed to hampered PM adoption at various levels within the US healthcare system. The results of the study highlight that clinicians and payers frequently fault each other for various implementation challenges, which is contributing to the overall adoption issues of PM and the community should focus on education of all stakeholders on the importance of PM and how it can directly lead to improved cancer outcomes.

Despite the current barriers, there is consensus that progress will be made to overcome today’s most significant implementation challenges over the next five years contingent on improved patient and provider education, increased institutional support, continued innovation and increased payer recognition of the value of PM. This study marks an opportunity to understand current viewpoints across stakeholder groups and to level set the community on the perceived barriers to PM. The ultimate goal is to help bolster the wide adoption of PM to result in improved cancer outcomes in the US.

## 2. Materials and Methods

### 2.1. Survey Methodology

We conducted a survey of medical oncologists, breast surgeons, thoracic surgeons, lab directors, payers, and cancer patients, to evaluate the precision medicine landscape and barriers in oncology. To assess the precision medicine landscape, five separate online surveys targeting each stakeholder groups in the U.S. were developed. To ensure high quality market research, the 653 survey respondents were recruited by market research vendors in compliance with industry standards in addition to outreach among Health Advances proprietary database of experts. All respondents were involved in precision medicine in some way—either based on their professional role in a hospital, lab, or payer organization, or as a patient who reported having cancer. Respondents answered questions that allowed for a quantitative picture of precision medicine in the U.S. All respondents were 21 or older at the time of the survey. Respondent demographics are displayed in Table 1. Survey development was informed by 60-min phone interviews with at least one of each stakeholder surveyed, except patients.

Patients. Respondents were required to have been diagnosed with metastatic cancer, or early-stage breast or lung cancer, in the past three years. 

Oncologists. Respondents were required to have been in practice more than 2 years but less than 36, spend more than 25% of their time in direct patient care, and manage more than 10 cancer patients per month. If the respondent reported seeing lung cancer patients they were required to have ordered biomarker testing for more than 10% of lung cancer patients.

Surgeons. Respondents were required to be either a thoracic or breast surgeon, been in practice for more than 2 years but less than 36, spend more than 25% of their time in direct patient care, and manage more than 5 cancer patients per month.

Payers. Respondents were required to have the title of medical director, clinical advisor, chief medical officer, laboratory benefits manager, or pharmacy director with experience working at payer organizations for >2 years at plans covering >10,000 lives. Respondents were required to be frequently or directly involved in policy, reimbursement, formulary placement, and coverage decisions for diagnostic testing and oncology. 

Lab Directors. Respondents were required to work in a clinical lab at a reference lab, academic hospital, or community hospital. They had to either oversee or supervise lab workflows and review/release test results and supervise the molecular section of the lab. They had to have processed over 10 cancer samples per month and test more than 10% for biomarkers on average.

### 2.2. Statistical Analysis of Barriers to PM across Stakeholders 

**Figure 1:**
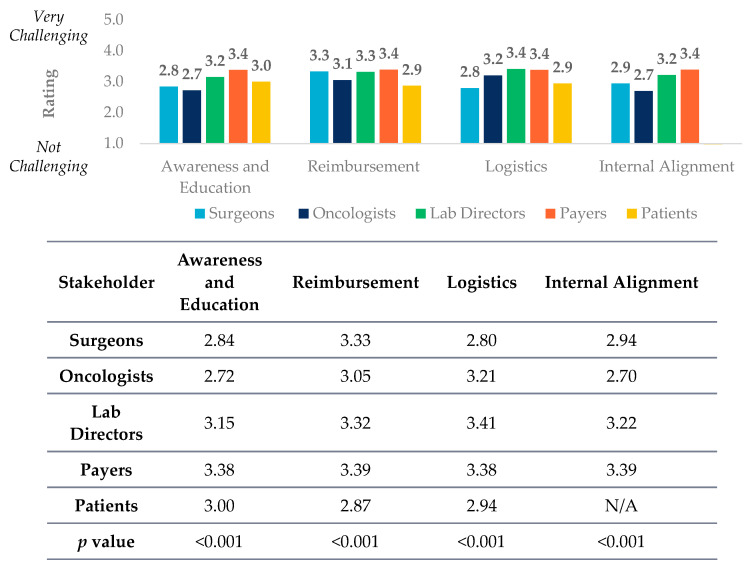
Lab directors, oncologists, surgeons, payers, and patients (*n* = 653) were asked the following question with different responses. Please rate how challenging the following factors are to biomarker testing for oncology targeted therapies on a scale of 1 to 5, where 1 is not challenging and 5 is very challenging. Responses were then clustered together in constructs around four different themes of awareness and education, reimbursement, logistics, and internal alignment. The average responses between respondents were found to be statistically significantly different (*p* value ≤ 0.001) for all constructs. Statistical analysis was done using a one-way ANOVA. Construct definitions can be found in methods.

In order to test if perceived barriers to biomarker testing were different across the five stakeholders, ANOVA was performed with a threshold value of *p* < 0.05 for statistical significance. Similar testing was also conducted to assess differences among respondents to compare stakeholders’ views of the barriers to PM. Despite seeing slightly different answer options, we assigned each answer into four different constructs for statistical analysis in Figure 1. Constructs included awareness and education challenges (which includes recognition of clinical utility), reimbursement, logistics, and internal alignment with definitions of questions included in each below. 

Awareness and Education Challenges Construct. Surgeon: Limited utility of multi target gene panel, Limited utility of comprehensive genomic profiling, Oncologists: Limited utility of multi target gene panel, Limited utility of comprehensive genomic profiling, Payers: Lack of internal expertise on biomarker tests, Lack of bandwidth to focus on biomarker tests, Lab directors: Difficulty interpreting multi target gene panel, Difficulty interpreting comprehensive genomic profiling, Patients: My doctor told me that biomarker testing was less studied among patients of my background, My oncologist didn’t seem to know much about biomarker tests, No one explained to me the importance of the test.

Reimbursement Construct. Surgeon: Lack of payer coverage of multi target gene panel for early-stage patient, Lack of payer coverage of multi target gene panel for late-stage patient, Lack of payer coverage of comprehensive genomic profiling for Early-stage patient, Lack of payer coverage of comprehensive genomic profiling for Late-stage patient, Oncologists: Lack of payer coverage of multi target gene panel for early-stage patient, Lack of payer coverage of multi target gene panel for late-stage patient, Lack of payer coverage of comprehensive genomic profiling for Early-stage patient, Lack of payer coverage of comprehensive genomic profiling for Late-stage patient, Payers: Over utilization by clinicians, Underutilization by clinicians, Lab directors: Delays in biomarker testing due to 14-day rule, Patients: I could not get insurance to cover the test.

Logistics Construct. Surgeon: Inability to test for biomarkers due to insufficient tissue, Oncologists: Inability to test for biomarkers due to insufficient tissue, Payers: Incorrect documentation for test reimbursement, Lab directors: Long turnaround times, Inability to test for biomarkers due to insufficient tissue, Patients: Biomarker testing was not offered at a location that was easily accessible for me, I did not have enough support needed to fill out the forms, I needed a second biopsy because not enough tissue was collected the first time, There was a long time from when I got my biomarker test to when I started treatment, I was worried about my genomic data being on an internet server

Internal Alignment Construct. Surgeon: Lack of internal alignment regarding whether there should be a reflex protocol, Lack of internal alignment regarding what should be on the reflex protocol, Oncologists: Lack of internal alignment regarding whether there should be a reflex protocol, Lack of internal alignment regarding what should be on the reflex protocol, Payers: Lack of internal consensus on appropriate biomarker tests for each cancer type, Lack of flexibility in coverage determinations for oncology tests and procedures, Lab directors: Lack of internal alignment regarding whether there should be a reflex protocol, Lack of internal alignment regarding what should be on the reflex protocol

## 3. Results and Discussion

A quantitative survey was conducted across five stakeholders including medical oncologists, surgeons, lab directors, payers, and patients to better understand the perceived barriers to PM utilization in the US. The outcome of the survey revealed that stakeholders are unaligned on the utilization challenges of PM testing stemming from a lack of education and awareness across healthcare professionals, payers, and patients. However, stakeholders were optimistic that the barriers to PM will decrease in the future with better alignment on the challenges to PM and education, and awareness of reimbursement and patient support.

### 3.1. Education, Awareness, and Reimbursement of PM Testing

All five stakeholders have significantly (*p* < 0.01) different viewpoints about education and awareness (which includes recognition of clinical utility) of different biomarker testing types. Payers view education and awareness challenges as a highest barrier in total amongst all of the stakeholder groups (Figure 1). 

**Figure 2:**
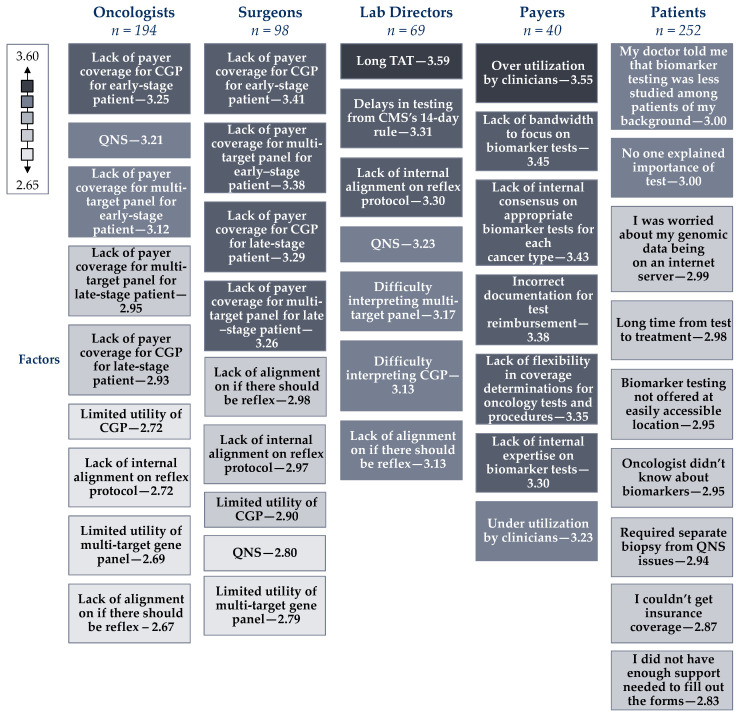
Lab directors, oncologists, surgeons, payers, and patients (*n* = 653) were asked the following question with different responses. Please rate how challenging the following factors are to biomarker testing for oncology targeted therapies on a scale of 1 to 5, where 1 is not challenging and 5 is very challenging. Responses are listed in order of largest to smallest challenge and shaded grey based on the legend from 2.60–3.60. Note: QNS = Quantity Not Sufficient defined as not enough specimen for a laboratory to perform the requested tests. TAT = Turn-around-time defined as the time taken from specimen collection to laboratory test results being reported. CMS = Centers for Medicare and Medicaid Services. CMS’s 14-day rule requires a clinical laboratory to seek reimbursement for testing ordered within 14 days of patients discharge.

Despite the small number of approved targeted therapies for early-stage breast and lung cancer as opposed to advanced disease, oncologists and surgeons still see value in ordering comprehensive genomic profiling (CGP) for early-stage breast and lung cancer patients (Table 2). This perspective is in contrast to payers which rank the utility of single gene, multi-target panels, and CGP all very closely, suggesting that payers may not understand which type of PM testing is best and why when considering the tumor type or stage of the cancer in question (Table 2). This is likely due to payers’ lack of bandwidth to focus on biomarker testing which was ranked as their second largest perceived barrier to PM (Figure 2).

There is a consensus among oncologists and payers, that of all testing types, CGP has the highest clinical utility in late-stage cancer across five tumor types included in the survey (lung, breast, ovarian, prostate, and bladder). This perception is noteworthy and may be a result of fewer targeted therapies specifically available for certain cancers such as ovarian and prostate, however, tumor agnostic targeted therapies still apply for these cancer types, for which CGP is the most effective testing strategy. Therefore, the data reflects a potential perception gap in utility of CGP (Table 2). 

Though the utility of CGP is widely accepted by both clinicians and payers, agnostic to cancer stage or tumor type, all stakeholders have significantly (*p* < 0.01) different viewpoints about reimbursement as a barrier to biomarker testing (Figure 1). Oncologists and surgeons view lack of reimbursement as the largest barrier to biomarker testing, specifically the lack of payer coverage for multi-gene panels and CGP for early-stage patients. Meanwhile, payers view over-utilization as the biggest individual challenge regarding biomarker testing (Figure 2). The views on the utility and reimbursement of different PM tests highlight the disconnect between clinicians and payers. The findings suggest that payers may not recognize the clinical utility of CGP for early-stage cancer patients, causing downward pressure on coverage and reimbursement. Meanwhile, discussions with payers have brought up concerns that clinicians may be ordering CGP too widely and payer groups are feeling increasing pressure to control costs. As the utilization of CGP in cancer care becomes more prevalent, payers indicate that reimbursement is likely to be the biggest hurdle that will prevent it from continuing to expand. 

To help alleviate the challenges discussed above the community should focus on of articulating the value of CGP to payers with targeted educational efforts such as they do with healthcare professionals (HCPs). Additionally, education of clinicians on the appropriate use cases of CGP is critical to the ecosystem of PM evolving in the near-term.

### 3.2. PM Patient Education and Access and Support Programs

Beyond payers and clinicians, there is also a disconnect between clinicians and patients on the relative level of awareness of biomarker testing and access. Many patients view themselves as having a high level of awareness and education of biomarker testing, however they are generally not aligned with clinicians in their understanding of the types of testing that they received, and their perceptions on the primary barriers to biomarker testing. 

**Figure 3:**
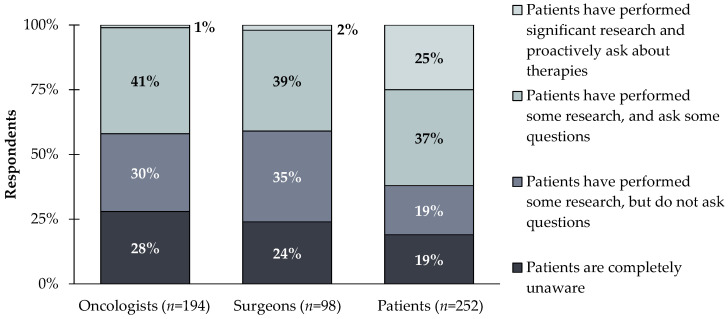
Oncologists and surgeons (*n* = 302) were asked the following question. Which of the following best describes your patients’ awareness of biomarkers and targeted therapies for their cancer? Patients (*n* = 242) were asked: Which of the following best describes your awareness of biomarker testing? 25% of patients believe they have performed significant research and proactively ask about therapies compared to the 1% of oncologists and 2% of surgeons.

Surgeons and medical oncologists assume most patients are uninformed about biomarker testing. In contrast, many patients show high confidence in their level of awareness. Only ~2% of clinicians compared to 25% of patients believe that patients have performed significant research and proactively ask about therapies (Figure 3). 

This divide is highlighted by patient perspectives on biomarker testing. Patients indicate they are aware of biomarker testing types, but less clear on the specific test that was ordered. Notably 54% of lung cancer, 70% of ovarian cancer patients, 58% of bladder cancer patients believed they were tested for a single gene. This compares to clinicians reporting that only 8–18% of lung cancer patients, 13–17% of ovarian cancer patients, and 7–17% of bladder cancer patients received single gene biomarker tests (range varies based on early- or late-stage), while 33% of breast cancer patients report they are unaware of what type of biomarker test they had (Table 3). Despite believing they are well-educated on biomarker testing, patients still view the largest barriers to PM as clinicians not educating them on when biomarker testing is indicated and the importance of the test results (Figure 2). 

**Figure 4:**
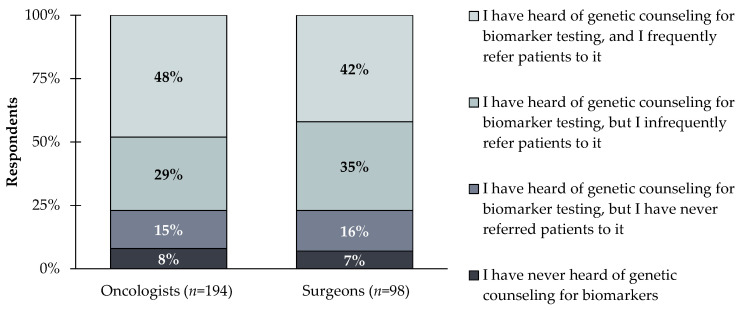
Oncologists and surgeons (*n* = 283) were asked the following question: Which of the following best describes your level of awareness of genetic counseling to support biomarker test ordering and results reporting? 77% of both oncologist and surgeon respondents report to being aware of genetic counseling for biomarker testing and referring patients at some point either frequently or infrequently.

An area where patients may be under-educated is related to cancer risk and understanding the difference between germline and somatic biomarker testing [10]. For germline testing in particular, broader incorporation of genetic counselling into multi-disciplinary cancer care delivery teams is key to enhancing patient understanding [11]. Over 50% of clinicians reported not frequently using genetic counseling, 8% of clinicians are unaware of genetic counseling for biomarker testing, and an additional 15% of clinicians are aware of genetic counselling for biomarker testing but having never referred patients to it (Figure 4). 

Not getting genetic counselors involved in discussions with patients about biomarker testing results can negatively impact patients. Genetic counselors can describe how biomarker testing can help guide treatment decisions and can help patients understand their own results. Though adding genetic counseling to cancer care teams is an effective healthcare delivery model shown to improve patient care [12], there are hurdles to implementing more use of genetic counselling. Challenges include a lack of genetic counselors with only 1.49 genetic counselors per 100,000 people in the US and disparities to access genetic counselling services by regional location, socioeconomic status, and cancer type [13]. One way to alleviate these issues is to increase the use of telehealth genetic counseling [14]. 

**Figure 5:**
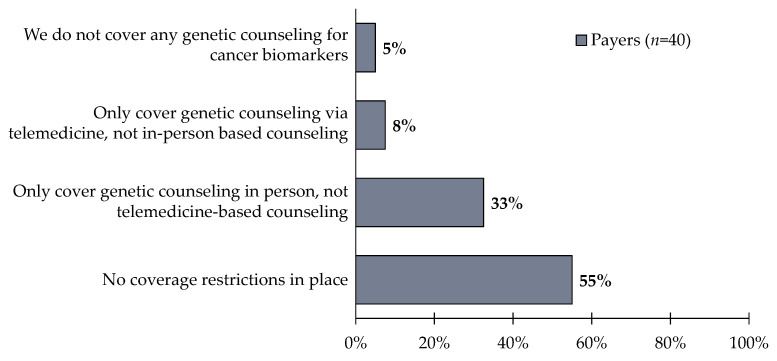
Payers (*n* = 40) were asked the following questions: Which of the following best describes your plan’s coverage of genetic counseling to support biomarker test ordering and results reporting? 55% of respondents reported having no coverage restrictions in place, but notably 33% of respondents report not covering telemedicine-based genetic counseling.

Despite COVID-19 accelerating the use of telemedicine, the widespread use of telehealth genetic counseling visits faces reimbursement issues. One notable issue is the Medicare policy which does not distinguish genetic counselors as providers eligible for reimbursement of any services, virtual or in-person [15]. Of the payer respondents surveyed in this study, 55% report having no coverage restrictions for genetic counseling; however, 33% reported only covering in-person genetic counseling, not telemedicine-based genetic counseling (Figure 5). Encouraging the reimbursement of telemedicine-based genetic counseling will lead to improved patient understanding of biomarker testing and how it impacts their cancer care.

**Figure 6:**
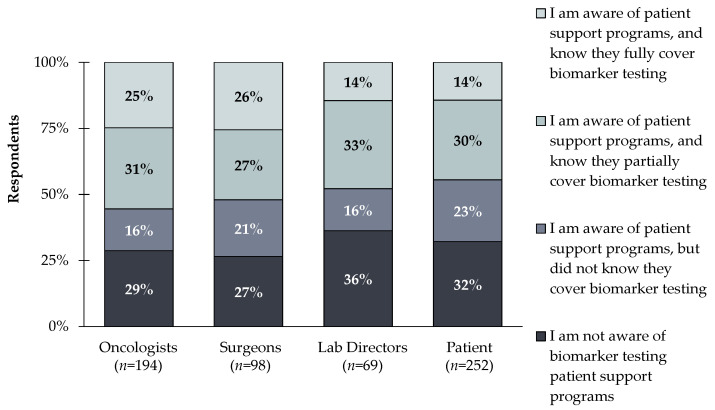
Oncologists, surgeons, lab directors, and patients (*n* = 613) were asked the following questions: Which of the following best describes your level of awareness of patient support programs for biomarker testing? A large amount of all respondents reported not being aware of biomarker testing patient support programs (29% of oncologists, 27% of surgeons, 26% of lab directors, and 32% of patients).

In addition to education, there is a lack of awareness of patient support programs that provide coverage for biomarker testing (e.g., Amgen’s BiomarkerAssist™ program). Only 26% and 14% of clinicians and patients respectively, reported being aware of patient support programs and knowing they fully cover biomarker testing. Meanwhile, 28% and 32% of clinicians and patients respectively, were not aware of biomarker testing patient support programs at all (Figure 6). Bringing awareness to these programs will ensure broader adoption of PM; especially since clinicians view reimbursement as the largest hurdle, but are unaware of assistance programs that can help bridge the gap.

The oncology community would be better served with an increased focus on patient education regarding the importance of biomarker testing and the clear implications it has in treatment as well as the educating the clinicians on the availability of support programs and encouraging the use of telehealth genetic counseling when available. 

## 4. Conclusions

**Figure 7:**
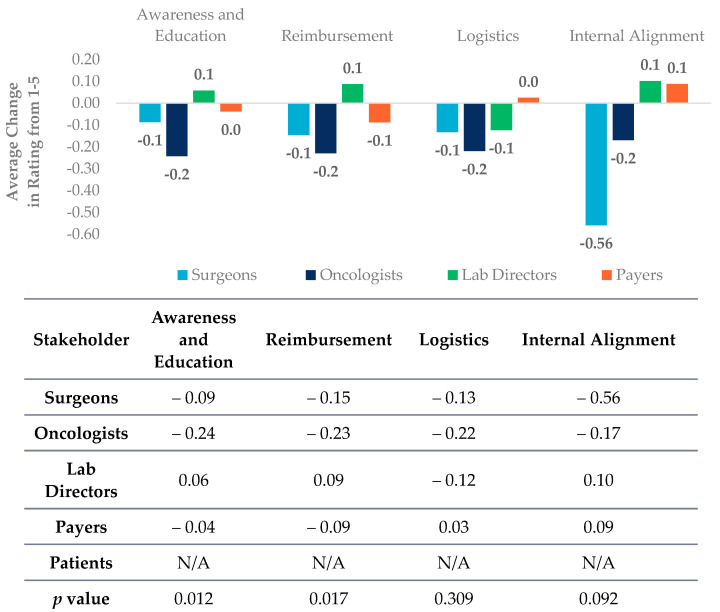
Lab directors, oncologists, surgeons, payers, and patients (*n* = 653) were asked the following question with different responses. Please rate how challenging the following factors are to biomarker testing for oncology targeted therapies today and in 5 years on a scale of 1 to 5, where 1 is not challenging and 5 is very challenging. The differences in lab directors, oncologists, surgeons, payers, and patients (*n* = 653) in responses to current barriers (Figure 1) and future barriers were assessed. The average responses between respondents were found to be statistically significantly with a *p* value < 0.05 for the constructs of awareness and education (*p* value = 0.012) and reimbursement (*p* value = 0.017) but not statistically significantly different for the constructs of logistics (*p* value = 0.309) and internal alignment (*p* value = 0.092). Statistical analysis was done using a one-way ANOVA. Construct definitions can be found in methods.

When probed on how the barriers to biomarker testing may change over the next five years oncologists, surgeons, and lab directors are overall optimistic that the challenge of the current barriers will decrease, specifically as it related to awareness and education and reimbursement (Figure 7). 

Interestingly, lab directors are less optimistic about the future of precision medicine. The decreased challenges to utility and reimbursement barriers relies on stakeholders aligning on the perceived challenges and bolstering awareness of clinical utility on different types of PM testing. This can be achieved through several different education strategies (1) payer education of the importance of PM in oncology to update coverage policies; (2) clinician education on the availability and reimbursement of PM testing; (3) multi-disciplinary team education on the available patient resources (4) incorporating lab directors, particularly pathologists as well as surgeons as part of the multidisciplinary team and molecular tumor boards. Collectively, by engaging and educating all stakeholders that are involved in patient care, the shared common goal of implementing precision medicine to improve patient care can be achieved.

## Figures and Tables

**Table 1:** Includes the demographics of all survey respondents delineated by stakeholder types. Stakeholders for this study include medical oncologists, surgeons, lab directors, payers, and cancer patients. Oncologists and surgeons are sometimes reported together and referred to as “clinicians” throughout the discussion.

Oncologist Demographics (*n* = 194)			
		Average Number of Patients/Month	Average Biomarker Tested
Cancer Types	Lung	45	87%
	Breast	58	81%
	Prostate	34	68%
	Ovarian	21	80%
	Bladder	20	70%
		Respondents	
Geographic Region	West	20%	
	Midwest	16%	
	South	33%	
	Northeast	31%	
Practice Type	Private Practice	46%	
	Academic Health System	38%	
	Community Health System	16%	
Surgeon Demographics (*n* = 98)			
		Average Number of Patients/Month	Average Biomarker Tested
Cancer Types	Lung	31	79%
	Breast	42	77%
		Respondents	
Geographic Region	West	12%	
	Midwest	17%	
	South	37%	
	Northeast	34%	
Practice Type	Private Practice	34%	
	Academic Health System	34%	
	Community Health System	33%	
Lab Director Demographics (*n* = 69)			
		Average Number of Patients/Month	Average Biomarker Tested
Cancer Types	Lung	163	62%
	Breast	211	65%
	Prostate	129	48%
	Ovarian	68	53%
		Respondents	
Geographic Region	West	29%	
	Midwest	23%	
	South	22%	
	Northeast	26%	
Practice Type	Private Practice	19%	
	Academic Health System	32%	
	Community Health System	49%	
Payer Demographics (*n* = 40)			
		Respondents	
GeographicRegion	West	13%	
	Midwest	13%	
	South	44%	
	Northeast	44%	
Geographic Reach	Single US State	28%	
	Regional (Multiple States)	40%	
	National Organization	32%	
Average Plan Size (Lives)	10,000–100,000	8%	
	100,000–1,000,000	18%	
	1,000,000–5,000,000	38%	
	5,000,000–10,000,000	15%	
	>10,000,000	40%	
Plan Breakdown of Lives Covered	Medicaid	12%	
	Commercial	33%	
	Medicare	54%	
Patient Demographics (*n* = 252)			
		Respondents	
Sex	Male	54%	
	Female	46%	
Cancer Types	Lung	25%	
	Breast	22%	
	Prostate	19%	
	Ovarian	6%	
	Bladder	27%	
Cancer Stage	Early-Stage	18%	
	Late-Stage	82%	
Geographic Region	West	32%	
	Midwest	23%	
	South	27%	
	Northeast	18%	
Ethnicity	White	93%	
	Latin American/Hispanic	2%	
	Black/African American	4%	
Insurance Type	Private Insurance	46%	
	Medicare	30%	
	Medicaid	13%	
	Veteran’s Affairs	<1%	

**Table 2:** Oncologists, surgeons, and payers (*n* = 332) were asked the following questions: Please rate the clinical utility of each test for early stage breast and lung cancer on a scale of 1 to 5, where 1 is not at all useful and 5 is extremely useful. Oncologists and payers. Please rate the clinical utility of each test for late-stage cancer on a scale of 1 to 5, where 1 is not at all useful and 5 is extremely useful. The highest-ranking test for each cancer type is shaded grey. Oncologists view CGP is having the highest clinical utility for all cancers. Payers mostly view multi-gene panel testing as having the highest clinical utility.

Respondent	PM Test Type	Lung Cancer		Breast Cancer		Late Stage Prostate Cancer	Late Stage Ovarian Cancer	Late Stage Bladder Cancer
		Early Stage	Late Stage	Early Stage	Late Stage			
Oncologists	*n*=	72		76		75	71	71
	Single Gene Test	2.8	2.7	3.0	3.1	2.6	2.9	2.7
	Multi-Gene Panel	3.6	3.7	3.8	3.8	3.5	3.6	3.6
	CGP	4.1	4.2	3.9	3.9	4.1	4.0	3.9
Surgeons	*n*=	38		60		N/A	N/A	N/A
	Single Gene Test	2.7	2.9	2.9	3.7	N/A	N/A	N/A
	Multi-Gene Panel	3.6	3.7	3.8	3.8	N/A	N/A	N/A
	CGP	3.7	3.7	3.8	4.0	N/A	N/A	N/A
Payers	*n*=	39		39		39	38	37
	Single Gene Test	3.3	3.2	3.4	3.3	3.2	3.3	3.1
	Multi-Gene Panel	3.7	3.5	3.3	3.6	3.5	3.8	3.5
	CGP	3.3	3.3	3.8	3.4	3.4	3.4	3.3

**Table 3:** Oncologists and surgeons (*n* = 292) were asked the following question: What percent of your patients receive the following biomarker test types? Patients (*n* = 211) were asked the following question: Which of the following test types sounds most like what you received from your doctor? How many genes were on the biomarker panel your doctor tested? Clinicians report using CGP testing (highlighted in grey) in a much higher percentage of patients than patients (shaded grey). A 5–33% of patients also report “I Don’t Know” for which type of biomarker testing they received.

Respondent	PM Test Type	Lung Cancer		Breast Cancer		Prostate Cancer		Ovarian Cancer		Bladder Cancer	
		Early Stage	Late Stage	Early Stage	Late Stage	Early Stage	Late Stage	Early Stage	Late Stage	Early Stage	Late Stage
Clinicians (oncologists and surgeons) *n* = 292	Single Gene Test	18%	10%	18%	13%	17%	8%	20%	13%	17%	7%
	Multi-Gene Panel	32%	29%	39%	36%	21%	22%	34%	30%	26%	19%
	CGP	50%	61%	40%	48%	54%	65%	47%	57%	43%	64%
Respondent	PM Test Type	Lung Cancer		Breast Cancer		Prostate Cancer		Ovarian Cancer		Bladder Cancer	
Patients *n* = 211	Single Gene Test	54%		33%		24%		70%		58%	
	Multi-Gene Panel	21%		16%		43%		0%		23%	
	CGP	16%		18%		18%		20%		14%	
	“I Don’t Know”	9%		33%		15%		10%		5%	

## Data Availability

Data are available from the corresponding author on reasonable request.
